# Gain-of-Function Mutant TP53 R248Q Overexpressed in Epithelial Ovarian Carcinoma Alters AKT-Dependent Regulation of Intercellular Trafficking in Responses to EGFR/MDM2 Inhibitor

**DOI:** 10.3390/ijms22168784

**Published:** 2021-08-16

**Authors:** Zih-Yin Lai, Kai-Yun Tsai, Shing-Jyh Chang, Yung-Jen Chuang

**Affiliations:** 1 Department of Medical Science & Institute of Bioinformatics and Structural Biology, National Tsing Hua University, Hsinchu 30013, Taiwan; laiziyin@gmail.com (Z.-Y.L.); a0956568498@gmail.com (K.-Y.T.); 2 Department of Obstetrics and Gynecology, Hsinchu MacKay Memorial Hospital, Hsinchu 30071, Taiwan

**Keywords:** p53^R248Q^ overexpression, epidermal growth factor receptor (EGFR), AKT, mouse double minute 2 homolog (MDM2), combination therapy

## Abstract

As the most common gene mutation found in cancers, p53 mutations are detected in up to 96% of high-grade serous ovarian carcinoma (HGSOC). Meanwhile, mutant p53 overexpression is known to drive oncogenic phenotypes in cancer patients and to sustain the activation of EGFR signaling. Previously, we have demonstrated that the combined inhibition of EGFR and MDM2-p53 pathways, by gefitinib and JNJ-26854165, exerts a strong synergistic lethal effect on HGSOC cells. In this study, we investigated whether the gain-of-function p53 mutation (p53^R248Q^) overexpression could affect EGFR-related signaling and the corresponding drug inhibition outcome in HGSOC. The targeted inhibition responses of gefitinib and JNJ-26854165, in p53^R248Q^-overexpressing cells, were extensively evaluated. We found that the phosphorylation of AKT increased when p53^R248Q^ was transiently overexpressed. Immunocytochemistry analysis further showed that upon p53^R248Q^ overexpression, several AKT-related regulatory proteins translocated in unique intracellular patterns. Subsequent analysis revealed that, under the combined inhibition of gefitinib and JNJ-26854165, the cytonuclear trafficking of EGFR and MDM2 was disrupted. Next, we analyzed the gefitinib and JNJ-26854165 responses and found differential sensitivity to the single- or combined-drug inhibitions in p53^R248Q^-overexpressing cells. Our findings suggested that the R248Q mutation of p53 in HGSOC caused significant changes in signaling protein function and trafficking, under EGFR/MDM2-targeted inhibition. Such knowledge could help to advance our understanding of the role of mutant p53 in ovarian carcinoma and to improve the prognosis of patients receiving EGFR/MDM2-targeted therapies.

## 1. Introduction

Epithelial ovarian cancer is the most prevalent (~90%) type of ovarian carcinoma [1] and could be further divided into subtypes, based on histopathology, gene alterations, and prognosis [2]. Among the five main subtypes, high-grade serous ovarian carcinoma (HGSOC) accounts for the highest proportion (~70%) [3]. HGSOC is known to have a high risk of recurrence and platinum-based chemotherapy drug resistance, due to genetic changes in tumor suppressor p53, encoded by *TP53* [4]. As one would expect, ovarian cancer has the highest rate of somatic *TP53* mutation among all cancers [5]. In fact, *TP53* is mutated in up to 96% of HGSOC, with arginine 248 (R248) being the most common mutated amino acid (p.Arg248Gln and p.Arg248Trp) [6].

Also, p53 acts as a transcription factor, with most mutations being located in its DNA-binding domain (DBD) [7,8]. In addition, it has been demonstrated that mutant p53 not only loses its activities, but also displays oncogenic gain-of-function (GOF), such as accelerating tumor progression and acquiring drug resistance [9]. Since mutant p53 is known to amplify receptor tyrosine kinase (RTK) signaling [10,11,12,13,14,15], the association between gain-of-function p53^R248Q^ and RTK-regulated signaling in HGSOC could be important for cancer progression. Among the RTK downstream signaling pathways, several studies have revealed that p53^R248Q^ promotes cell migration and proliferation, through AKT signaling [16,17]. However, how the R248Q mutation of p53 affects AKT-dependent carcinogenesis has not been elucidated. Excessive activation of AKT signaling can mediate a variety of cellular processes, including cell cycle dysregulation, proliferation, and drug resistance, all of which are considered to be hallmarks of cancer [18]. In addition, it has been elucidated that activated AKT translocates to various organelles, where AKT phosphorylates substrates or interacts with other factors, to regulate a complex network of processes [19]. For instance, AKT phosphorylates MDM2 on serine 166 and serine 186, to initiate MDM2′s nuclear translocation. MDM2 then acts to promote p53 ubiquitination for degradation and reduce p53 transcriptional activities [20]. Similarly, the phosphorylation of EGFR’s serine 229, by AKT, also promotes the nuclear translocation of EGFR [21], which acts as a co-transcription factor of multiple genes, regulating cell proliferation and tumor angiogenesis, resulting in enhanced drug resistance and poor patient outcomes [22]. It should be noted that whether the R248Q mutation of p53 affects AKT-dependent molecular trafficking has not been determined.

The taxane- and platinum-based chemoresistance in ovarian cancer is known to be associated with its high mortality rates [23]. Moreover, numerous studies have indicated that up to 60% of epithelial ovarian cancer displays EGFR overexpression. However, the treatment of ovarian cancers with the anti-EGFR agent gefitinib alone generates a limited response [24]. When gefitinib is used together with platinum-based chemotherapy drugs, the combination therapy would increase the overall response rate in patients with relapsed ovarian cancer [25]. However, such combination therapy has exhibited limited efficacy in other clinical trials [26]. Such finding suggests a need to modify the treatment scheme.

Previous studies have shown that up to 80% of ovarian serous borderline tumors exhibit MDM2 overexpression [27], while the expression of MDM2 is notably low in benign ovarian tumors or normal ovaries [28]. Biomarker analysis has found that the co-expression of p53 and MDM2 is associated with poor outcomes in epithelial ovarian cancer patients [29]. Furthermore, our previous study demonstrated that the combined inhibition of EGFR and MDM2-p53 pathways in HGSOC could generate a strong synergistic anti-cancer effect [30]. These findings led us to explore whether the R248Q mutation of p53 would affect the drug efficacy and the underlying mechanisms by which EGFR and MDM2-p53 interact, to produce a synergistic effect.

In this study, we first investigated how p53^R248Q^ overexpression altered the characteristics of HGSOC. We then explored how the R248Q mutation of p53 regulated the drug responses and synergistic effect, under the combined inhibition of EGFR and MDM2.

## 2. Materials and Methods

### 2.1. Cell Culture

The serous ovarian cancer cell line OVCAR3 (p53^R248Q^ mutation) was obtained from Dr. Chih-Long Chang (MacKay Memorial Hospital). The *TP53* status was annotated according to the International Agency for Research on Cancer (IARC) *TP53* database (http://p53.iarc.fr/; accessed date: 30 September 2019). The cells were cultured at 37 °C with 5% CO_2_ in RPMI-1640 medium (Gibco) supplemented with 10% fetal bovine serum, 100 units/mL penicillin, 100 μg/mL streptomycin, 2.5 μg/mL amphotericin B, 2 mg/mL sodium bicarbonate, 3.57 mg/mL HEPES and 0.11 mg/mL sodium pyruvate.

### 2.2. Drugs and Reagents

Gefitinib (LC Laboratories, Woburn, MA, USA) and JNJ-26854165 (AdooQ BioScience, Irvine, CA, USA) were dissolved in DMSO and stored at −20 °C. EGF (Sigma-Aldrich, St. Louis, MO, USA) was dissolved in 0.2-μm-filtered 10 mM acetic acid and stored at −20 °C.

### 2.3. Plasmids Construction and Overexpression

The wild-type p53 (wtp53) and p53^R248Q^ mutation (p53^R248Q^) plasmids were constructed in house. The full-length wtp53 and p53^R248Q^ sequences were made from A2780 and OVCAR3 cells. Total cDNA was amplified by using KAPA HiFi DNA polymerase (KAPABIOSYSTEMS). The primer sequences were the following: 5′-CTCGAGGCCTGAGGTTGGCTCTGACT-3′ and 5′-GAATTCGGCACAAACACGCACCTCAA-3′. The PCR products were constructed into pJET1.2/blunt cloning vector (Fermentas) and sub-cloned into pEGFP-N1 vector (Addgene). The final plasmids were sequence verified. Cells were transfected with plasmids at a final concentration of 1 μg/mL using jetPRIME (Polyplus) following the manufacturer’s protocols.

### 2.4. Western Blot Analysis

The detailed experimental procedure was performed as previously described [31]. The anti-p-EGFR (#2220), p-AKT (#4060), AKT (#4691), p-MAPK (#4370), MAPK (#4695), and p21 (#2947) antibodies were obtained from Cell Signaling Technology. The anti-EGFR (GTX628888), p53 (GTX102965) and GAPDH (GTX627408) antibodies were obtained from GeneTex. Each experiment was performed in triplicate and repeated at least three times.

### 2.5. Immunocytochemistry (ICC)

OVCAR3 cells were seeded on sterile glass coverslips prior to the experiments. The detailed experimental procedure was performed as previously described [31]. The anti-EGFR (green signal, #2232), AKT (#4691), and FOXO3a (#2497) antibodies were obtained from Cell Signaling Technology. The anti-EGFR (purple signal, GTX628888) and MDM2 (GTX110608) antibodies were obtained from GeneTex. The anti-mutant p53 (OP29) antibody was obtained from Merck. Phalloidin and Hoechst 33342 were obtained from Invitrogen. Each experiment was performed in triplicate and repeated at least three times.

### 2.6. Cell Viability Assay

OVCAR3 cells were seeded in 96-well plates at a density of 5 × 10^3^ cells/well. Cells were transfected with empty plasmid only (1 μg/mL) or with the p53^R248Q^ construct (1 μg/mL) for 18 h and then treated with drugs of different concentrations for 48 h. Following the manufacturer’s protocols, 10 μL cell counting kit-8 (CCK-8, Dojindo) cell proliferation reagent was added to 96-well plates and incubated at 37 °C and 5% CO_2_ for 1 to 2 h. The optical densities to reflect cell viability were determined by absorbance wavelength at 450 nm. The IC_50_ values were calculated by GraphPad Prism 6 (GraphPad software, San Diego, CA, USA). Each experiment was performed in triplicate and repeated at least three times.

### 2.7. Analysis of Combination Index

The combination index (CI) values were calculated by CompuSyn calculation software as described by Chou and Talalay [32]. First, the cell viability assay data were converted into the fraction affected (Fa) under specified drug treatment conditions. Next, the Fa values of single agents or drug combinations were uploaded into CompuSyn and generated the CI values, the Fa-CI plots and the isobolograms as instructed by the user manual.

### 2.8. Statistics

The data were processed by SigmaPlot v.10 (Systat Software) and expressed as the means ± SEM (standard error of the mean). The statistical significance was determined by *p* values < 0.05 using Student’s *t*-test.

## 3. Results

### 3.1. Overexpression of p53^R248Q^ was Associated with p-AKT Expression in OVCAR3 Cells

**Figure 1:**
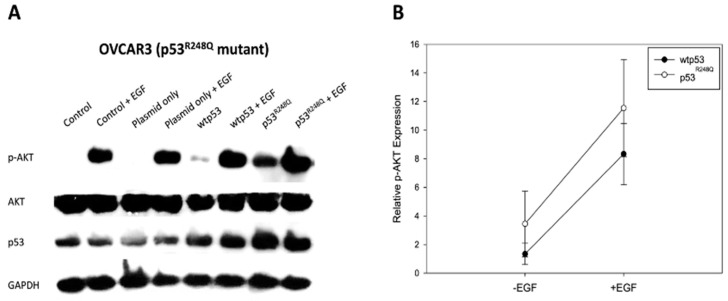
p53^R248Q^ resulted in regulation of p-AKT. (**A**) The level of p-AKT was increased after p53^R248Q^ overexpression in OVCAR3 cells. It should be noted that OVCAR3 cells were transiently transfected with either empty plasmid (1 μg/mL), wtp53 (1 μg/mL), or p53^R248Q^ (1 μg/mL) for 24 h and then stimulated with EGF for 15 min. GAPDH expression was measured to serve as a loading control. (**B**) The band intensity of the immunoblots was quantified using ImageJ software. The relative intensities of p-AKT expression were normalized with the control group. (wtp53: wild-type p53 plasmid; p53^R248Q^: p53^R248Q^ mutation plasmid).

Since EGFR (a member of the RTK family) is aberrantly overexpressed and activated in ovarian cancer [33,34], we first investigated whether p53^R248Q^ overexpression affected EGFR downstream signaling in OVCAR3 cells (HGSOC cell line with p53^R248Q^). In particular, AKT has been shown to be involved in p53^R248Q^-related cancer progression [16,17]. Therefore, we examined the EGFR downstream marker AKT, under the condition of p53^R248Q^ overexpression, by Western blot. We stimulated pre-starved OVCAR3 cells with epidermal growth factor (EGF), to activate the EGFR signaling. As expected, we found that the augmented expression of the p53^R248Q^ groups in OVCAR3 cells had a higher expression level of p-AKT (Figure 1A). Furthermore, we also observed the slight increasing trend of the statistical results in the p53^R248Q^ and p53^R248Q^ + EGF groups compared to the wtp53 and wtp53 + EGF groups (Figure 1B). Further, siRNA assay validated the association between p53 mutation and p-AKT level (Figure S1). Taken together, these data suggested that the R248Q mutation of p53 augmented p-AKT signaling in OVCAR3 cells.

### 3.2. p53^R248Q^ Overexpression and EGF Stimulation Resulted in Similar Cytonuclear Trafficking of AKT, EGFR, MDM2, and FOXO3a

**Figure 2:**
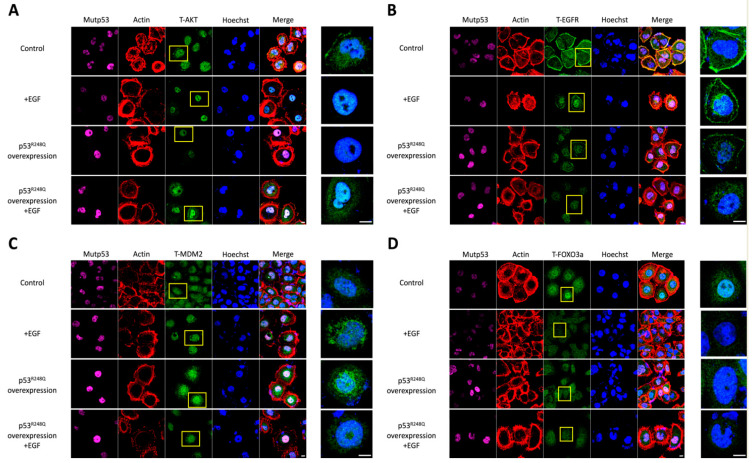
p53^R248Q^ overexpression and EGF stimulation altered the intracellular localization patterns of AKT, EGFR, MDM2 and FOXO3a. In this experiment, we first transfected OVCAR3 cells with or without the p53^R248Q^ expression plasmid (1 μg/mL) for 24 h and then serum-starved the cells for another 24 h. Before confocal imaging, we stimulated the cells in the presence or absence of EGF (50 ng/mL) for 15 min. That is, the following four conditions were analyzed: the “control” group, in which the cells were serum-starved only; the “+EGF” group, in which the cells were treated with EGF after serum starvation; the “p53^R248Q^ overexpression” group, in which the cells were transfected with p53^R248Q^ before serum starvation; and the “p53^R248Q^ overexpression + EGF” group, in which the cells were transfected with p53^R248Q^, serum-starved, and then treated with EGF. Since the cells were only transiently transfected with p53^R248Q^, we immunostained the cells with an anti-mutant p53 antibody to ensure that the transfected cells overexpressed p53^R248Q^. (**A**) Confocal image analysis revealed that most AKT translocated into the nucleus completely after EGF stimulation or p53^R248Q^ overexpression. However, when the cells were overexpressed with p53^R248Q^ and then stimulated with EGF, some AKT remained in the cytoplasm. (**B**) In the control group, most EGFR accumulated on the cell membrane, while some EGFR distributed evenly in the cytoplasm. After EGF stimulation or p53^R248Q^ overexpression, EGFR appeared to converge and translocate towards the nucleus. When overexpressed with p53^R248Q^ and then stimulated with EGF, EGFR could not be detected on the cell membrane. (**C**) In the control group, MDM2 could be detected in the cytoplasm. After EGF stimulation, MDM2 dynamically converged around the nucleus. Such phenomena could also be observed after overexpression of p53^R248Q^. With both p53^R248Q^ overexpression and EGF stimulation, MDM2 translocated into the nucleus. (**D**) FOXO3a, which mostly accumulated in the nucleus in the control group, was evenly distributed in the cytoplasm after treatment with either EGF stimulation, p53^R248Q^ overexpression, or both. The cells were immunostained with anti-AKT antibody, anti-EGFR antibody, anti-MDM2 antibody, anti-FOXO3a antibody (shown in green), and anti-mutant p53 antibody (shown in purple); actin in red and DNA in blue were shown by Phalloidin staining and Hoechst staining, respectively. The right images were enlarged view of the yellow boxed region. The scale bar represented 10 μm.

AKT signaling is known to mediate the intracellular trafficking of various receptors and regulatory proteins. We thus investigated how the R248Q mutation of p53 altered the intracellular molecular trafficking of EGFR, MDM2, and FOXO3a, in association with AKT (Figure 2).

We found that, after either EGF stimulation or p53^R248Q^ overexpression, most of the AKT translocated into the nucleus completely. However, when p53^R248Q^ overexpression was combined with EGF stimulation, some AKT remained in the cytoplasm (Figure 2A). Consistent with past studies, our data implied that AKT entered the nucleus in response to growth factors, to exert regulatory activities [35,36,37]. Thus, we continued to investigate how AKT translocation correlated with the intracellular trafficking of EGFR and MDM2.

Similar, but different, from the aforementioned findings, after EGF stimulation or p53^R248Q^ overexpression, both EGFR and MDM2 appeared to converge and translocate to the periphery of the cell nucleus (Figure 2B,C). In comparison to the control group, we also observed some EGFR still localized on the cell membrane, while most of the MDM2 translocated to the periphery of the cell nucleus. Interestingly, when p53^R248Q^ overexpression was combined with EGF stimulation, the EGFR located on the cell membrane and the MDM2 clustering around the nucleus, were not detected, and most of the MDM2 translocated into the cell nucleus.

On the other hand, it has been reported that FOXO3a, a member of the forkhead box O (FoxO) transcription factor families and a tumor suppressor, accelerates its nuclear export when it is phosphorylated by nuclear AKT [38]. Therefore, we speculated that this tumorigenic marker was affected by p53^R248Q^. Based on our data, we found that FOXO3a, which mostly localized in the nucleus in the control group, distributed uniformly in the cytoplasm, under the following conditions: after EGF stimulation, with p53^R248Q^ overexpression, and p53^R248Q^ overexpression combined with EGF stimulation (Figure 2D). These findings suggested that the R248Q mutation of p53 promoted tumorigenesis-related AKT signaling, by affecting molecular intracellular trafficking.

### 3.3. Combined Blockade by Gefitinib and JNJ Attenuated EGFR and MDM2 Cytonuclear Trafficking

The comparable translocation patterns of EGFR and MDM2 implied that the molecular cytonuclear trafficking may relate to the synergistic effect of combined EGFR and MDM2 inhibition that has been reported in our previous study [30]. In this present study, we again used gefitinib (EGFR tyrosine kinase inhibitor) and JNJ-26854165 (referred to as JNJ; MDM2 E3 ubiquitin ligase domain inhibitor) to determine whether such combined inhibition would alter the intracellular localization patterns of EGFR and MDM2.

**Figure 3:**
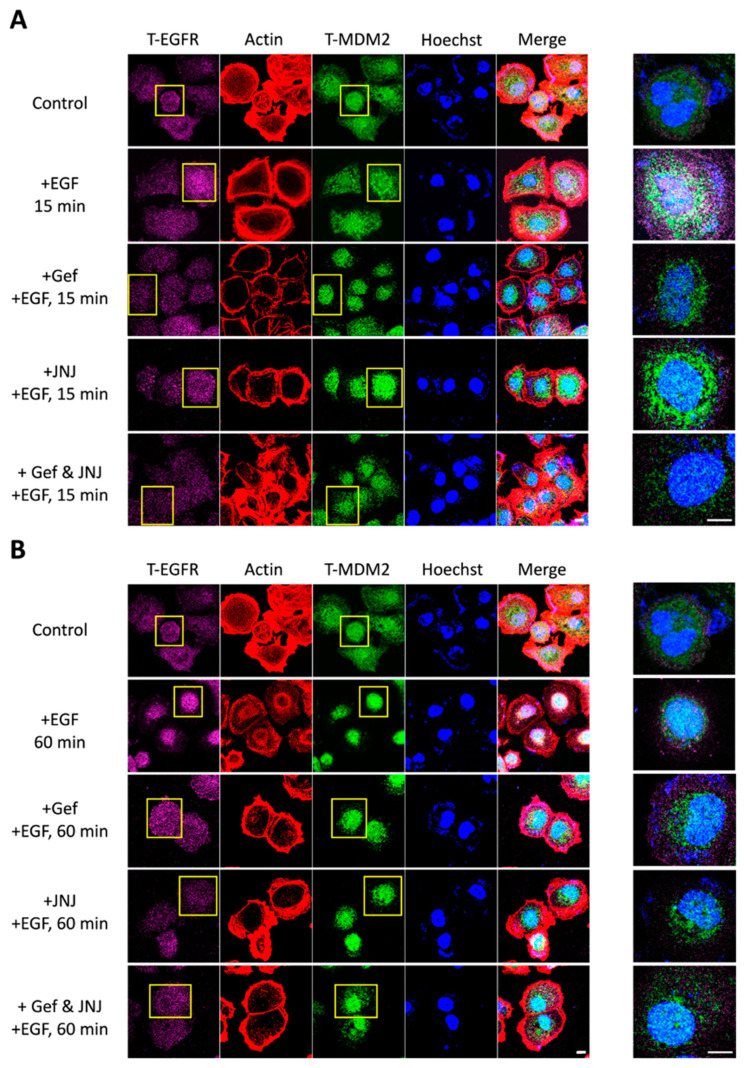
Cytonuclear trafficking of EGFR and MDM2 would be disrupted under combined inhibition of EGFR and MDM2 by gefitinib and JNJ. OVCAR3 cells were serum-starved while treated with or without the inhibitors under different conditions (i.e., 14.4 μM gefitinib, 1.4 μM JNJ, or 1 μM gefitinib + 1 μM JNJ; the IC_50_ concentration was derived from our previous study [30]) for 24 h. The cells were then stimulated with EGF (50 ng/mL) for 0 (control), 15 or 60 min. (**A**) Confocal image analysis demonstrated that EGFR and MDM2, which were initially distributed in the cytoplasm, converged and translocated in sync to the periphery of the nucleus after 15 min of EGF stimulation. However, the convergence pattern of EGFR was disrupted after 24 h of treatment with gefitinib, JNJ, or their combination. In comparison, the convergence pattern of MDM2 was disrupted only under the combined treatment of gefitinib and JNJ. (**B**) After 60 min of EGF stimulation, EGFR and MDM2 translocated completely into the nucleus. With combined treatment of gefitinib and JNJ for 24 h, some MDM2 could be detected again in the cytoplasm. The cells were immunostained with anti-EGFR antibody (shown in purple) and anti-MDM2 antibody (shown in green); actin in red and DNA in blue were shown by Phalloidin staining and Hoechst staining, respectively. The right images were enlarged view of the yellow boxed region. The scale bar represented 10 μm. (Gef: gefitinib; JNJ: JNJ-26854165).

By immunocytochemistry staining analysis, we first reconfirmed that both EGFR and MDM2 would translocate from the cytoplasm to the nucleus, in accordance with each other over time, after EGF stimulation (Figure S2). Next, we found that such nuclear convergence of EGFR was attenuated under treatment with gefitinib, JNJ, or both. Interestingly, the nuclear convergence of MDM2 was only disrupted under the combination treatment of gefitinib and JNJ (Figure 3A,B).

These results indicated that the combined inhibition of gefitinib and JNJ could prevent the converging nuclear translocation of EGFR and MDM2, which might regulate further signaling and oncogenic activity.

### 3.4. R248Q Mutation of p53 Increased the Sensitivity of EGFR and MDM2 Inhibitors

To further explore the impact of p53^R248Q^ on the cellular response to combination therapy for HGSOC, we evaluated the anti-cancer efficacy of gefitinib and JNJ in OVCAR3 cells with different p53 statuses. As shown in Table S1, the combined inhibition of EGFR and MDM2 significantly reduced the IC_50_ values compared to the single agent treatment. Interestingly, the IC_50_ values of gefitinib and JNJ alone reduced to 78% (from 33.74 to 26.47 μM) and 66% (from 21.83 to 14.35 μM), when the cells were overexpressing p53^R248Q^. On the other hand, the combined inhibition resulted in a slight reduction (from 8.57 to 8.18 μM) in IC_50_.

**Figure 4:**
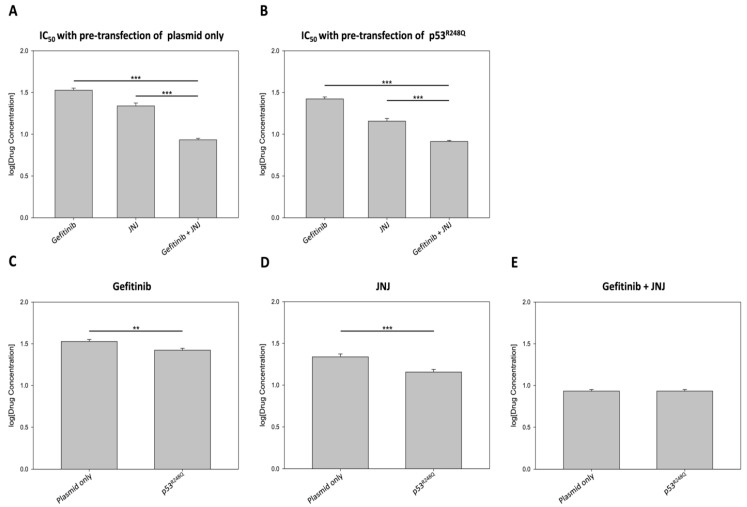
Overexpression of p53^R248Q^ in OVCAR3 cells increased sensitivity to gefitinib or JNJ. In this experiment, OVCAR3 cells were pre-transfected with either empty plasmid (1 μg/mL) or p53^R248Q^ (1 μg/mL) for 18 h and then exposed to the inhibitors alone or in combination for another 48 h. The doses of gefitinib and JNJ ranged from 5 to 40 μM and 1 to 36 μM, respectively. The concentrations of gefitinib and JNJ in combination were prepared in a one-to-one ratio, with doses ranging from 0.5 to 12 μM. The effects on cell proliferation were assessed by CCK-8 assay; the half maximal inhibitory concentration (IC_50_) values (in log10) were calculated by GraphPad Prism 6. (**A**) In the absence or (**B**) in the presence of p53^R248Q^ overexpression, the combined treatment of gefitinib and JNJ significantly reduced the IC_50_ values for each agent in OVCAR3 cells (*** *p* < 0.001 vs. gefitinib and JNJ). (**C**,**D**) The sensitivities to gefitinib or JNJ alone increased significantly in p53^R248Q^-overexpressing cells (** *p* < 0.01; *** *p* < 0.001). (**E**) No significant difference was found when the combination treatment of gefitinib and JNJ was applied to the plasmid-only transfected cells and the p53^R248Q^-overexpressing cells.

For a more intuitive comparison, we summarized the data as in Figure 4, to highlight the significant reduction in effective drug concentration, under combined inhibition. A synergistic effect was observed, whether cells were pre-transfected with the p53^R248Q^ plasmid or not (Figure 4A,B). Interestingly, gefitinib and JNJ alone were more effective when OVCAR3 cells were pre-transfected with the p53^R248Q^ plasmid (Figure 4C,D). However, the combined inhibition effect remained similar, regardless of whether the OVCAR3 cells were pre-transfected with the p53^R248Q^ plasmid or not (Figure 4E). Taken together, these results suggested that the R248Q mutation of p53 increased the sensitivity of OVCAR3 cells to EGFR and MDM2 inhibition, to various degrees.

### 3.5. R248Q Mutation of p53 Decreased the Synergistic Lethal Effect of Gefitinib and JNJ

To better evaluate the synergistic effects exerted by combined inhibition in different p53 statuses, we adapted the well-known Chou and Talalay’s combination index (CI) method, to perform in-depth analysis. It should be noted that a CI value less than one is defined as drug synergism [32,39].

**Figure 5:**
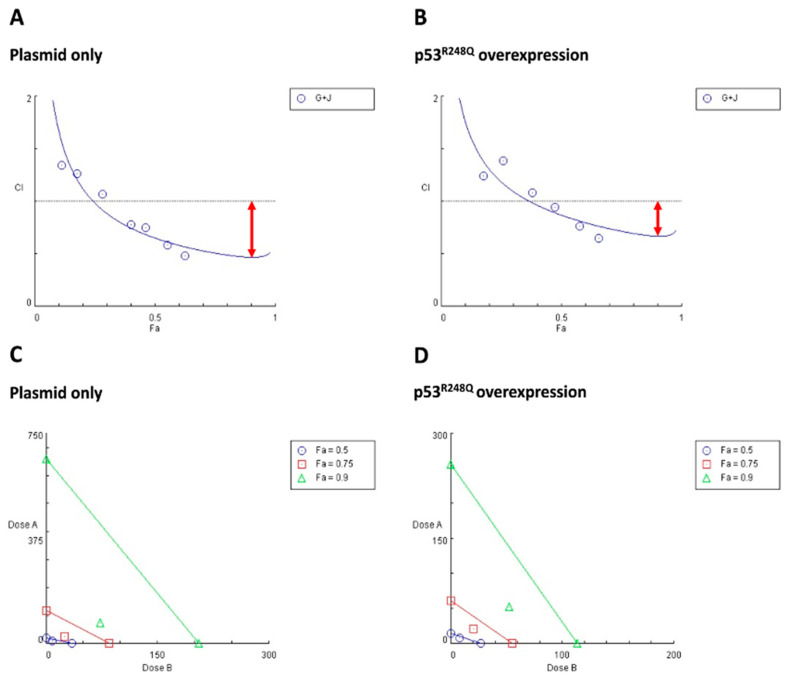
Synergistic effects of gefitinib and JNJ on OVCAR3 cells with different p53 statuses were shown in Fa-CI plots and isobolograms. Based on the Chou and Talalay methods, the combination index (CI) values were calculated using CompuSyn software to determine the degree of synergistic effectiveness of the drug combination. The analysis revealed that overexpression of p53^R248Q^ reduced the synergistic effect of gefitinib and JNJ. (**A**,**B**) In the Fa-CI plots, the fraction affected (Fa; from zero to one) represented the ratio of dead cells-to-total cells in response to drug treatments. Circled symbols indicated the CI values of each Fa. The CI values provided a quantitative definition of synergistic (CI < 1), additive (CI = 1), and antagonistic (CI > 1) effects derived from the drug combination. (G + J: gefitinib + JNJ) (**C**,**D**) In the isobolograms, the individual doses of JNJ (represented by *x*-axis) and gefitinib (represented by *y*-axis) that achieved 90% (Fa = 0.9), 75% (Fa = 0.75), and 50% (Fa = 0.5) inhibitory effects were indicated by green lines, red lines, and blue lines, respectively. Symbols (ED_90_ was marked as green triangles; ED_75_ was marked as red squares; ED_50_ was marked as blue circles) above the lines, on the lines, and below the lines represented antagonistic, additive, and synergistic effects, respectively. When the symbols were located below and distant from the lines, it represented strong synergism.

The CI analysis revealed that, in the absence of the overexpression of p53^R248Q^, the area below the CI = 1 line was larger than that of the p53^R248Q^ overexpression group (Figure 5A,B). We also generated isobolograms, to quantify the drug synergism at different effective doses (EDs). In comparison to the p53^R248Q^ overexpression group, the symbols (colored as specified) were farther from the corresponding colored lines, and the CI values were also lower in the absence of the overexpression of p53^R248Q^ (Table S2, Figure 5C,D). The CI values and the synergism grading were summarized in Table S2.

In OVCAR3 cells overexpressing p53^R248Q^, the drug combination displayed slight synergism (CI = 0.87) at ED_50_, but changed to moderate (CI = 0.72) and enhanced synergism (CI = 0.67) at ED_75_ and ED_90_. Interestingly, OVCAR3 cells without p53^R248Q^ overexpression showed enhanced synergism (CI = 0.65) at lower doses (ED_50_). This finding implied that a higher dose of gefitinib and JNJ (ED_90_) was required to reach synergism in OVCAR3 cells overexpressing p53^R248Q^. In contrast, a lower dose of drug combination (i.e., ED_50_) exerted synergism in OVCAR3 cells without p53^R248Q^ overexpression. Such data supported that the overexpression of p53^R248Q^ may attenuate the synergistic lethal effect of gefitinib and JNJ.

### 3.6. MAPK and p21 Regulated the Effects of Single and Combined Treatment of Gefitinib and JNJ

To provide more clues about the regulatory effect of p53^R248Q^ on the observed differential sensitivity to gefitinib and JNJ, we analyzed the expression profiles of MAPK and p21 (a p53 target gene associated with cell cycle and apoptosis pathways), under different conditions of gefitinib and JNJ concentration and combination.

**Figure 6:**
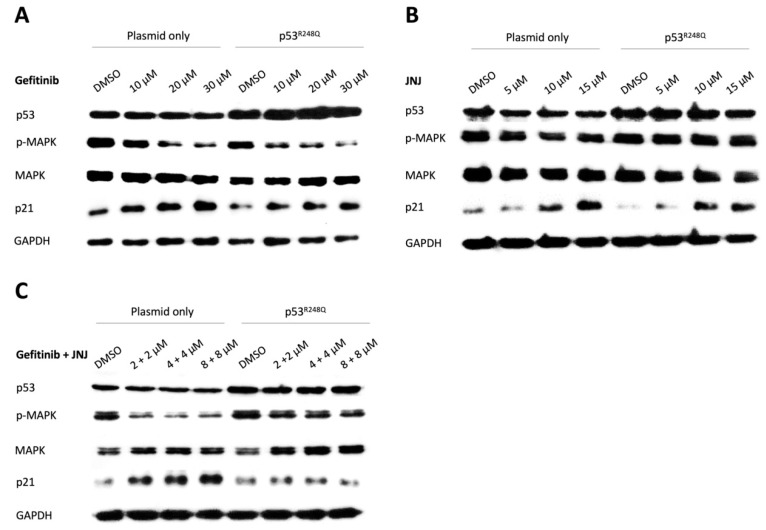
MAPK and p21 regulated the effects of single and combined treatment of gefitinib and JNJ. OVCAR3 cells were pre-transfected with either empty plasmid or p53^R248Q^ plasmid before treatment with various doses of gefitinib (10, 20 or 30 μM), JNJ (5, 10, or 15 μM), or gefitinib + JNJ (2 + 2, 4 + 4, or 8 + 8 μM) for 24 h. (**A**) The level of p-MAPK was significantly reduced with increasing doses of gefitinib. The dosage-dependent inhibition of p-MAPK displayed an even more profound effect in the p53^R248Q^-overexpression group. The level of p21 was upregulated in both groups with increasing doses of gefitinib. The dosage-dependent upregulation of p21 was not significantly different between the two groups. (**B**) With increasing doses of JNJ, the level of p-MAPK decreased slightly in the plasmid-only group, while sustained level of p-MAPK was detected in the p53^R248Q^-overexpression group. The level of p21 was upregulated significantly with increasing doses of JNJ. Despite the lower level of p21 in the DMSO control of the p53^R248Q^-overexpression group, the dosage-dependent upregulation of p21 was greater in the p53^R248Q^-overexpression group than in the plasmid-only group. (**C**) With increasing doses of the gefitinib–JNJ combination, the level of p-MAPK significantly decreased in the plasmid-only group, but not in the p53^R248Q^-overexpression group. Moreover, the level of p21 increased with a profound effect in the plasmid-only group, but not in the p53^R248Q^-overexpression group. GAPDH expression was measured to serve as a loading control.

As expected, the enhanced expression of p53 was detected, which served to validate the overexpression of p53^R248Q^ after transfection. Next, the results revealed that p-MAPK was significantly reduced by gefitinib, in a dose-dependent manner (Figure 6A). Moreover, at a dose of 10 μM, gefitinib exerted a slight MAPK-reduction effect in p53^R248Q^-overexpressing cells compared to the cells without overexpression (Figure S3A). We also found that the expression level of p21 was significantly increased by JNJ, in a dosage-dependent manner. Upon examination and comparison of the two DMSO control groups, we found that the expression level of p21 was lower in p53^R248Q^-overexpressing cells (Figure 6B). However, with increasing JNJ dose, p21 increased significantly (approximately 25-fold) in p53^R248Q^-overexpressing cells, compared to the cells without overexpression (approximately 3.5-fold) (Figure S3B). Strikingly, the effects were reversed when JNJ was combined with gefitinib (Figure 6C, Figure S3C,D).

These results implied that in the presence of p53^R248Q^ overexpression, the augmented sensitivity to gefitinib or JNJ, and the weakened synergistic effect of the combined treatment, might be associated with the alternating expression of MAPK and p21.

## 4. Discussion

In this study, we provided new evidences to highlight the effects of the R248Q mutation of p53 on intercellular trafficking and the cellular responses to EGFR/MDM2-targeted inhibition in HGSOC.

**Figure 7:**
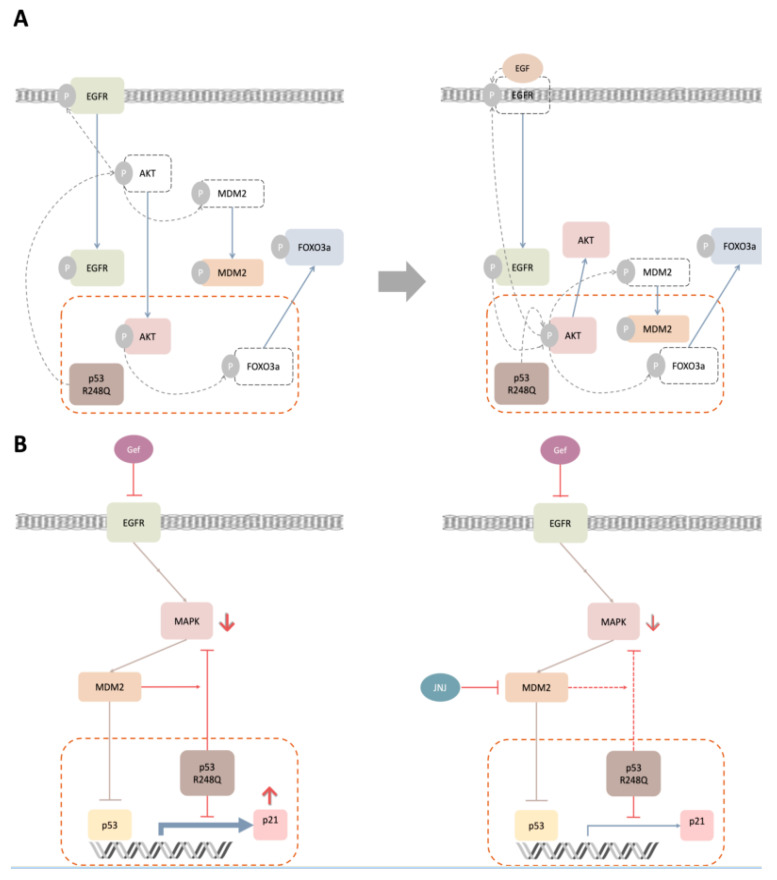
Hypothetical models to integrate molecular trafficking and synergistic lethal effects of gefitinib and JNJ with p53^R248Q^ overexpression. (**A**) This model scheme depicted the signal transduction and molecular trafficking under p53^R248Q^ overexpression and/or EGF stimulation in OVCAR3 cells. (**B**) This model scheme depicted the molecular regulation under gefitinib and/or JNJ treatments in OVCAR3 cells with p53^R248Q^ overexpression.

Mutant p53 is generally considered to accelerate carcinogenesis, by carrying out a dominant-negative effect on wild-type p53 and by manifesting gain-of-function activities [40]. In this study, our findings implied that the phosphorylation of AKT would be affected in OVCAR3 cells via p53^R248Q^ overexpression (Figure 1). For the effects of enhanced p-AKT signaling in HGSOC, we first speculated that the subcellular localization of AKT, as well as AKT-related molecular trafficking, may be altered under the influence of p53^R248Q^. This hypothesis was supported by the evidences that nuclear AKT, induced by various growth factors and stimuli, has been shown to promote tumorigenesis, by controlling cell cycle progression, promoting cell survival and DNA repair, and counteracting apoptosis [19]. In this study, we had further examined the trafficking patterns of AKT, EGFR, MDM2, and FOXO3a, upon EGF stimulation and p53^R248Q^ overexpression (Figure 2). Our data revealed that these molecules’ trafficking patterns, under p53^R248Q^ overexpression, exert a comparable effect to that of EGF stimulation. Based on our findings, we proposed a hypothetical model of molecular trafficking in response to EGF stimulation and p53^R248Q^ overexpression (Figure 7A).

In this model scheme, the R248Q mutation of p53 could amplify the phosphorylation of AKT, similarly to the stimulation of EGF. The resulting p-AKT would further phosphorylate EGFR and MDM2, to facilitate their converging nuclear translocation. Furthermore, nuclear AKT would further phosphorylate FOXO3a, to dispel it from the nucleus. Surprisingly, the combined p53^R248Q^ overexpression and EGF stimulation did not lead to the augmentation of nuclear AKT. In contrast, such combined treatment could promote more EGFR and MDM2 to translocate towards or into the nucleus. To the best of our knowledge, such phenomena have not been reported previously. We speculated that the AKT phosphorylation, induced by EGFR activation and p53^R248Q^ overexpression, resulted in antagonism, while the dual phosphorylation, induced by nuclear AKT and EGF on EGFR, led to synergism. The difference in the translocation pattern of MDM2 may arise from the differential signaling of nuclear AKT and cytoplasmic AKT. The underlying mechanisms shall be further explored in future studies.

To view this study in a broader context, evidences accumulated over the past 20 years have helped to characterize the function of nuclear AKT, which phosphorylates various transcription factors, including members of the FOXO transcription factor families [41]. The phosphorylated FOXO3a would be expelled from the nucleus and weaken its transcriptional capacity. As a result, the expression level of its target gene, such as *p21*, was reduced [42] (Figure 6). Nuclear EGFR has been shown to act as a co-transcription factor, alongside STAT3, E2F1, and STAT5, to initiate the transcription of several cell cycle regulatory genes, such as *Cyclin D1*, *Aurora A*, and *Myc* [22]. Nuclear MDM2 mainly diminishes the cellular level of p53 and inhibits its transcriptional activity [20,43,44]. Therefore, the nuclear localization of AKT, EGFR, and MDM2, and the exclusion of nuclear FOXO3a, have been linked with enhanced drug resistance, poor prognosis, and unfavorable overall survival in various cancers [22,45,46,47,48].

To date, the combination of paclitaxel- and platinum-based drugs is a standard first-line treatment for ovarian cancer. However, chemoresistance to these drugs may lead to an unfavorable 5-year survival rate in advanced patients [49]. To overcome drug resistance, we suggested a novel combination therapy strategy, with EGFR and MDM2 inhibition, in our previous study [30]. In the present study, we further identified that the disruption of cytonuclear trafficking, by the dual inhibition of EGFR and MDM2, might contribute to the synergistic effect of gefitinib and JNJ (Figure 3). In addition, we demonstrated that the R248Q mutation of p53 enhanced the efficacy of gefitinib and JNJ alone, but reduced the synergistic effect of their combination (Figure 4 and Figure 5). Our data further validated this observation, by showing the differential effect of p-MAPK inhibition and the differential effect of p21 induction, under different gefitinib and JNJ treatment conditions (Figure 6). These findings suggested that the degree of synergism of gefitinib and JNJ depended not only on the cytonuclear trafficking of the signaling mediators, but also on the mutation of p53.

Based on our findings, the proposed model (Figure 7B) underlined the mechanistic roles of p-MAPK and p21 under the treatment of gefitinib and JNJ, in the presence of p53^R248Q^ overexpression. It should be noted that MDM2 is known to ubiquitinate the pro-apoptotic transcription factor FOXO3a via activated MAPK signaling [50]. As expected, with p53^R248Q^ overexpression, gefitinib may cooperate with p53^R248Q^, to reduce p-MAPK. Our model further explained how the reduction in p-MAPK was primarily influenced by p53^R248Q^, and secondarily influenced by MDM2. Hence, the relevant cell signaling would be disrupted by JNJ, while gefitinib, in the presence of p53^R248Q^ overexpression, could not exert its expected effects on p-MAPK and p21 (Figure 7B). This model has the potential to revise the therapeutic approach for cancer treatment, which suggests the use of gefitinib or JNJ alone if the R248Q mutation of p53 is detected in HGSOC patients; if the R248Q mutation of p53 is not detected, the combination of gefitinib and JNJ should be recommended. While additional work is needed to confirm these predictions, we foresee that further understanding the relationship between the p53 mutation and the altered regulatory mechanisms, could enhance our knowledge of ovarian carcinogenesis and the implementation of precision cancer medicine.

## Data Availability

No data available.

## Supplementary Materials

The following are available online at https://www.mdpi.com/article/10.3390/ijms22168784/s1.

## Abbreviations

CI: combination index; ED: effective dose; ED_50, 75, 90_: 50%, 75%, 90% effective dose, respectively; EGF: epidermal growth factor; EGFR: epidermal growth factor receptor; Fa: fraction of affected cells; FOXO3a: forkhead box O3; HGSOC: high-grade serous ovarian carcinoma; IC_50_: half-maximal inhibitory concentration; JNJ: JNJ-26854165, also known as Serdemetan; MAPK: mitogen-activated protein kinase, also known as ERK; MDM2: mouse double minute 2 homolog; p53^R248Q^: p53 R248Q mutant; p-AKT: phosphorylated AKT; PI3K: phosphoinositide 3-kinase; p-MAPK: phosphorylated MAPK; RTK: receptor tyrosine kinase
