# Supplementary material for: Gain-of-Function Mutant TP53 R248Q Overexpressed in Epithelial Ovarian Carcinoma Alters AKT-Dependent Regulation of Intercellular Trafficking in Responses to EGFR/MDM2 Inhibitor
Source: Int J Mol Sci. 2021 Aug 16;22(16):8784. doi: 10.3390/ijms22168784 (PMC8395913; doi:10.3390/ijms22168784)
Supplement: Supplementary file 1 [file ijms-22-08784-s001.zip › ijms-1298945-supplementary.pdf]

Table S1. IC_{50} values of OVCAR3 cells with different p53 status to EGFR and/or MDM2 inhibitors.

Cell Line	Cell Status	Drug Treatment	IC_{50} (μM)
OVCAR3	Pre-transfected with empty plasmid for 18 hours	Gefitinib	33.74
		JNJ	21.83
		Gefitinib + JNJ	8.57
	Pre-transfected with $p53^{R248Q}$ for 18 hours	Gefitinib	26.47
		JNJ	14.35
		Gefitinib + JNJ	8.18

Table S2. Combination index (CI) values and synergism grading of combined gefitinib and JNJ treatment at different effective doses (ED_{50}, ED_{75} and ED_{90}).

CI values of specified effective dose (ED) for OVCAR3 cells with different p53 statuses were summarized. The synergism grading of combined inhibition was based on the grading standard defined by Chou and Talalay [39]. CI values from 0.85 to 0.9, 0.7 to 0.85 and 0.3 to 0.7 indicated slight synergism, moderate synergism and synergism, expressed as "+", "++" and "+++", respectively.

Cell Line	Cell Status	CI Values and Synergism Grading		
		ED_{50}	ED_{75}	ED_{90}
OVCAR3	Pre-transfected with empty plasmid for 18 hours	0.65<br>+++	0.51<br>+++	0.47<br>+++
	Pre-transfected with $p53^{R248Q}$ for 18 hours	0.87<br>+	0.72<br>++	0.67<br>+++

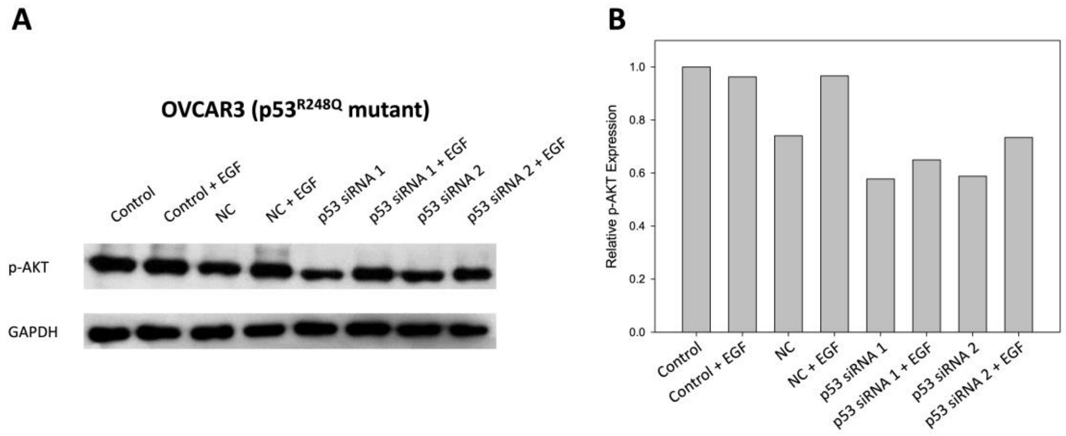

Figure S1. p53^{R248Q} resulted in regulation of p-AKT.

(A) The level of p-AKT was reduced after transfection with p53 siRNA in OVCAR3 cells. It should be noted that serum-starved OVCAR3 cells was transiently transfected with negative control siRNA (25 nM) or p53 siRNA (25 nM) for 24 hours and then stimulated with EGF for 15 minutes. GAPDH expression was measured to serve as a loading control.

(B) The band intensity of the immunoblots was quantified using ImageJ software. The relative intensities of p-AKT expression were normalized with the control group.

(NC: negative control siRNA)

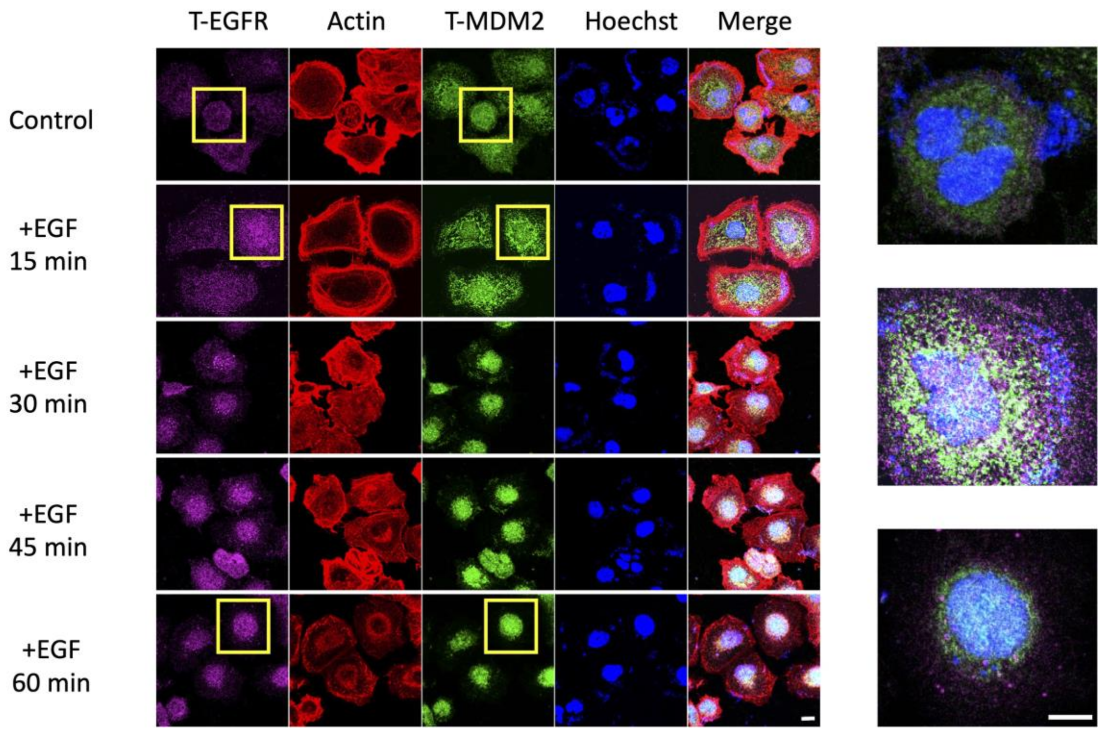

Figure S2. Time-dependent cytonuclear trafficking patterns of EGFR and MDM2. EGFR and MDM2 moved in a convergent manner from the cytoplasm to the nucleus over time after EGF stimulation. The right images were enlarged view of the yellow boxed region. The scale bar represented 10 μm.

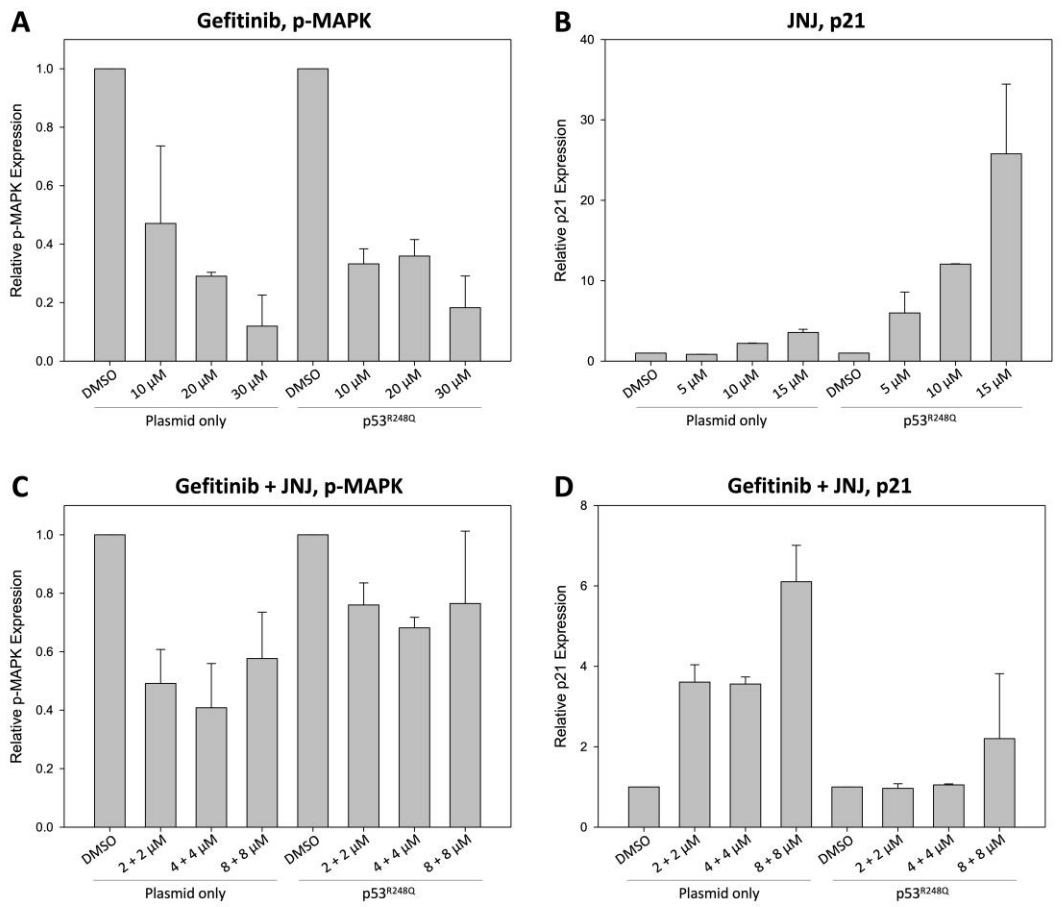

Figure S3. Quantification graphs of immunoblots in Figure 6.

The band intensity of the immunoblots was quantified using ImageJ software. The relative intensities of p-MAPK or p21 expression were normalized with the DMSO control group.
